# Splicing defects in rare diseases: transcriptomics and machine learning strategies towards genetic diagnosis

**DOI:** 10.1093/bib/bbad284

**Published:** 2023-08-14

**Authors:** Robert Wang, Ingo Helbig, Andrew C Edmondson, Lan Lin, Yi Xing

**Affiliations:** Center for Computational and Genomic Medicine, Children’s Hospital of Philadelphia, Philadelphia, PA 19104, USA; Genomics and Computational Biology Graduate Program, University of Pennsylvania, Philadelphia, PA 19104, USA; The Epilepsy NeuroGenetics Initiative, Children’s Hospital of Philadelphia, Philadelphia, PA 19104, USA; Division of Neurology, Children’s Hospital of Philadelphia, Philadelphia, PA 19104, USA; Department of Biomedical and Health Informatics, Children’s Hospital of Philadelphia, Philadelphia, PA 19104, USA; Department of Neurology, University of Pennsylvania, Philadelphia, PA 19104, USA; Center for Computational and Genomic Medicine, Children’s Hospital of Philadelphia, Philadelphia, PA 19104, USA; Department of Pediatrics, Division of Human Genetics, Children’s Hospital of Philadelphia, Philadelphia, PA 19104, USA; Department of Pathology and Laboratory Medicine, University of Pennsylvania, Philadelphia, PA 19104, USA; Raymond G. Perelman Center for Cellular and Molecular Therapeutics, Children’s Hospital of Philadelphia, Philadelphia, PA 19104, USA; Center for Computational and Genomic Medicine, Children’s Hospital of Philadelphia, Philadelphia, PA 19104, USA; Department of Biomedical and Health Informatics, Children’s Hospital of Philadelphia, Philadelphia, PA 19104, USA; Department of Pathology and Laboratory Medicine, University of Pennsylvania, Philadelphia, PA 19104, USA

**Keywords:** splicing, rare disease, variant interpretation, diagnostics, RNA sequencing, machine learning

## Abstract

Genomic variants affecting pre-messenger RNA splicing and its regulation are known to underlie many rare genetic diseases. However, common workflows for genetic diagnosis and clinical variant interpretation frequently overlook splice-altering variants. To better serve patient populations and advance biomedical knowledge, it has become increasingly important to develop and refine approaches for detecting and interpreting pathogenic splicing variants. In this review, we will summarize a few recent developments and challenges in using RNA sequencing technologies for rare disease investigation. Moreover, we will discuss how recent computational splicing prediction tools have emerged as complementary approaches for revealing disease-causing variants underlying splicing defects. We speculate that continuous improvements to sequencing technologies and predictive modeling will not only expand our understanding of splicing regulation but also bring us closer to filling the diagnostic gap for rare disease patients.

## INTRODUCTION

One of the defining quests in the era of genomic medicine has been to comprehensively delineate the genetic basis of rare diseases. Rare diseases collectively impact more than 300 million people worldwide, with over 7000 distinct conditions catalogued to date [1, 2]. Despite the often chronic and progressive nature of these conditions, having a definitive molecular diagnosis can reduce prognostic uncertainty, facilitate access to appropriate healthcare resources, relieve emotional burden on patients and their families, and present the first step toward precision therapies. To this end, identifying causative genes and variants underlying rare disease phenotypes is critical for improving patient outcomes as well as advancing biomedical knowledge.

Since 2009, the advent of next-generation sequencing (NGS) strategies, which enable comprehensive ascertainment of both rare and common genetic variation at remarkably low costs, has revolutionized the way we investigate rare diseases. NGS approaches for DNA analysis, such as exome and genome sequencing, have rapidly accelerated our capacity to identify disease-causing variants in both known and novel disease genes. In fact, it has been estimated that nearly three times as many rare disease genes have been discovered using NGS compared with previous methods, which have traditionally relied on positional cloning and linkage analysis [3]. Today, NGS-based DNA sequencing is routinely used as the primary approach for diagnosing rare diseases in the clinic and has supplanted older technologies. While there has been unquestionably positive development in using NGS to advance our understanding of rare disease etiology, the diagnostic success rate for detecting causal variants still remains far from complete. For example, the UK 100 000 Genomes Project showed that ~25% of previously undiagnosed patients with suspected rare disease could be diagnosed by genome sequencing [4]. Overall, the molecular diagnostic rate of NGS-based DNA sequencing is estimated to be between 25 and 40% across various patient cohorts, leaving a considerable diagnostic gap that remains to be filled [5, 6].

One of the primary challenges underlying this gap concerns variant interpretation—that is, the number of variants revealed by NGS substantially exceeds our current ability to interpret their functional and clinical impact. Existing clinical workflows for variant interpretation focus almost exclusively on variants within or adjacent to protein-coding regions, such as nonsense, frameshift and missense variants, as well as variants that disrupt canonical splice sites (i.e. the GT/AG, GC/AG or AT/AC dinucleotides flanking each intron [7]) [8]. As a result, these workflows will often overlook rare variants with functional, and possibly pathogenic, consequences on gene regulation, especially those falling in non-coding regions of the genome. In particular, one class of variants that continues to elude detection in clinical practice are those that disrupt normal patterns of pre-messenger RNA (mRNA) splicing. It has been estimated that roughly 15–30% of variants causing inherited diseases have the potential to disrupt splicing, and these variants are not restricted to those falling within canonical splice sites [9]. Missense, nonsense and synonymous variants within coding exons, as well as intronic variants, can disrupt pre-mRNA splicing and cause disease [10, 11]. Given growing evidence that splicing aberrations frequently contribute to rare disease etiology, developing clinical variant interpretation workflows that more carefully consider splice-altering variants is expected to improve genetic diagnosis rates. In practice, however, detecting and interpreting genomic variants that impact splicing remains challenging due to our incomplete understanding of the ‘splicing code’ and its multiple layers of regulation [12, 13].

This review aims to summarize a few recent technological advances that have improved our ability to discover pathogenic splice-altering variants in patient genomes. To better contextualize the significance of these advances, we will provide an overview of the currently known mechanisms that govern pre-mRNA splicing and how these mechanisms can be perturbed in the context of rare genetic disorders. We will summarize recent applications of RNA sequencing for rare disease investigation and discuss some of the challenges and future opportunities that lurk within this space. Moreover, we will review some of the major computational splicing prediction tools released to date and examine how these tools have complemented RNA sequencing (RNA-seq) approaches to reveal disease-causing variants that impact splicing.

### Pre-mRNA splicing and its dysregulation in rare diseases

**Figure 1:**
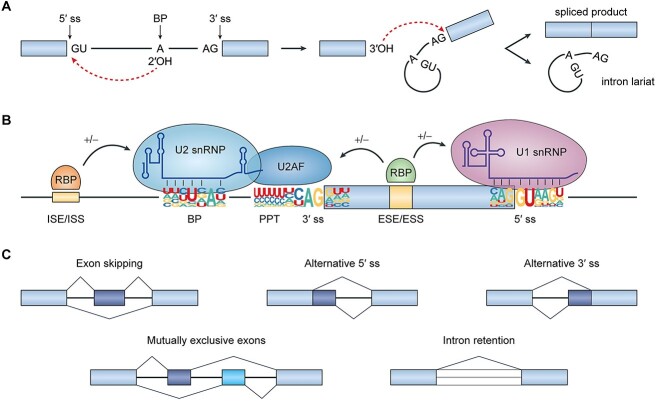
Pre-mRNA splicing and its regulation. (**A**) During splicing, introns are removed from pre-mRNA transcripts by a two-step process. In the first step, the 2′-hydroxyl group of the branchpoint adenosine makes a nucleophilic attack on the 5′ splice site, which cleaves the upstream exon from the intron and generates an intron lariat intermediate. In the second step, the 3′-hydroxyl group at the 5′ splice site attacks the 3′ splice site, which results in ligation of the two exons and release of the intron lariat. (**B**) Pre-mRNA splicing is regulated by a vast protein-RNA interaction network involving *cis* elements along the pre-mRNA as well as *trans* factors that recognize these elements. The core splicing signals include the 5′ splice site (which has a conserved GU dinucleotide), the 3′ splice site (which has a conserved AG dinucleotide), branchpoint sequence and polypyrimidine tract. U1 snRNP recognizes the 5′ splice site, and U2 snRNP recognizes the branchpoint sequence. U2AF proteins engage with the 3′ splice site and polypyrimidine tract. Additional *cis* regulatory elements (splicing enhancers and silencers) further modulate splicing through interactions with *trans* acting RBPs. Sequence logos were generated using enoLOGOS webtool [15] based on position weight matrices from Zhang *et al*. [16] (**C**) Five basic patterns of alternative splicing. Abbreviations: ss, splice site; OH, hydroxyl; BP, branchpoint; PPT, polypyrimidine tract; ESE, exonic splicing enhancer; ESS, exonic splicing silencer; ISE, intronic splicing enhancer; ISS, intronic splicing silencer; RBP, RNA-binding protein; snRNP, small nuclear ribonucleoprotein; U2AF, U2 auxiliary factor.

Pre-mRNA splicing is a biochemical process in which introns of a nascent pre-mRNA molecule are removed, and exons are joined together to form a mature mRNA molecule [14]. This process consists of two steps (Figure 1A). First, an internal adenosine (i.e. the branchpoint) makes a nucleophilic attack on the 5′ end of the intron, which cleaves the upstream exon from the intron and generates an intron lariat intermediate. Next, the end of the upstream exon attacks the 3′ end of the intron, which joins together the two exons and releases the intron lariat.

Despite the seemingly simple nature of this process, pre-mRNA splicing requires both precise and efficient identification of exons, which can be separated by more than 100 kb of intronic sequences along a pre-mRNA transcript [17]. To this end, eukaryotic splicing is orchestrated by an extensive network of protein-RNA interactions involving *cis* elements embedded within the pre-mRNA as well as *trans* factors that recognize these elements [18]. At the core of this network is the spliceosome, a dynamic macromolecular complex whose components (e.g. U1 and U2 snRNPs) recognize and interact with the core sequences required for splicing, including the 5′ and 3′ splice sites, the branchpoint sequence and the polypyrimidine tract. These interactions are further modified by other auxiliary *cis* elements located within exons and introns that can either enhance (i.e. ‘splicing enhancer elements’) or inhibit (i.e. ‘splicing silencer elements’) splicing. This is accomplished through interactions with *trans* acting regulatory factors known as RNA-binding proteins (RBPs), whose expression levels and activities can vary across different biological contexts, such as cell type or developmental stage (Figure 1B) [19].

During pre-mRNA splicing, alternative choices of exons and splice sites can arise depending on spatiotemporal context, resulting in a phenomenon commonly referred to as alternative splicing. More than 90% of human protein-coding genes are known to undergo alternative splicing, which includes exon skipping, alternative 5′ and 3′ splice site usage, mutually exclusive exons and intron retention (Figure 1C) [20, 21]. The resulting mRNA isoforms can have distinct regulatory properties in the cell (e.g. altered localization and stability) and be translated into proteins with unique structures and functions [22].

**Figure 2:**
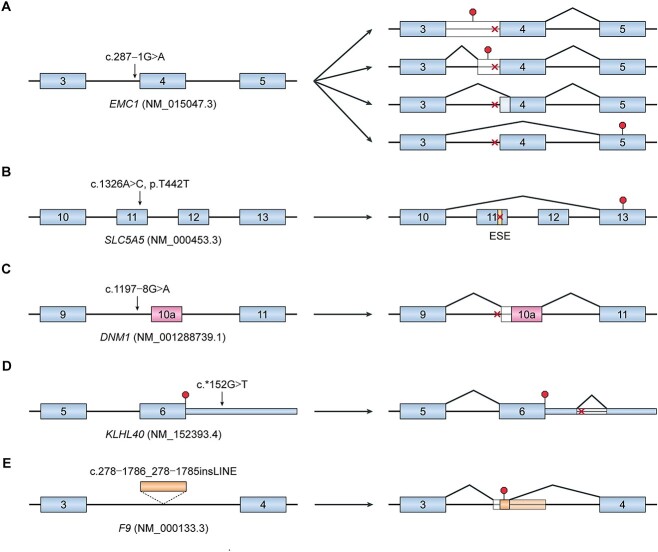
Splicing defects underlying rare diseases. (**A**) A point mutation disrupting the 3′ splice site of *EMC1* intron 3 results in multiple splicing consequences, including exon 4 skipping, intron 3 retention and activation of cryptic 3′ splice sites in intron 3 and exon 4. This mutation was reported to cause severe global developmental delay and progressive cerebellar atrophy. (**B**) In a case of congenital hypothyroidism, a synonymous variant in *SLC5A5* exon 11 disrupts an exonic splicing enhancer element, resulting in skipping of both exons 11 and 12. (**C**) Across multiple cases of developmental and epileptic encephalopathy, a *de novo* intronic variant upstream of *DNM1* exon 10a was found to create a cryptic 3′ splice site that extends the beginning of exon 10a by 6 bp. (**D**) A point mutation in the 3′ UTR of *KLHL40* creates a cryptic 5′ splice site, leading to the removal of an aberrant 78 bp intron from the 3′ UTR and provoking nonsense-mediated mRNA decay of *KLHL40* transcripts. This mutation was found in a patient diagnosed with nemaline myopathy. (**E**) In a case of hemophilia B, a ~6 kb LINE-1 insertion in *F9* intron 3 leads to the exonization of a 137 bp chimeric pseudoexon that uses a 5′ splice site within the inserted sequence and a pre-existing 3′ splice site located upstream of the insertion site. Red octagons represent stop codons, and red crosses represent mutations. Abbreviations: ESE, exonic splicing enhancer; LINE, long interspersed nuclear element.

Given the complexity of *cis* elements and *trans* factors needed for regulating pre-mRNA splicing, it is rather unsurprising that this biochemical process is highly susceptible to genomic variants implicated in disease. For many rare genetic disorders, splicing dysregulation predominantly occurs at the *cis* acting level [23], where mutations in a gene lead to splicing aberrations in the gene itself. In particular, mutations that disrupt 5′ and 3′ splice site dinucleotides are known to cause many rare diseases and are usually picked up by standard diagnostic workflows [12, 23]. A meta-analysis of 18 950 disease-relevant splice-site dinucleotide variants in the Human Gene Mutation Database (release 2021.4) suggests that variants affecting the 5′ splice site dinucleotide are reported at approximately the same frequency as variants affecting the 3′ splice site dinucleotide (54.7 and 45.3% for 5′ and 3′ splice sites, respectively) [24]. Splice site mutations are usually known to induce exon skipping, although other consequences may arise depending on local sequence context (Table 1) [25–27, 29–31]. For example, a point mutation disrupting the 3′ splice site of *EMC1* intron 3 was discovered in an individual with severe global developmental delay and progressive cerebellar atrophy [31]. Based on reverse transcriptase-polymerase chain reaction (RT-PCR) analysis of patient skeletal muscle, this point mutation was found to not only induce skipping of exon 4, but also cause retention of intron 3 as well as activation of two different cryptic 3′ splice sites (Figure 2A).

**Table 1:** Representative examples of *cis* acting splicing mutations underlying diagnosed cases of rare diseases

Disease	Mutation	Mechanism	Effect on splicing
Proximal myopathy, no ophthalmoplegia [25]	NM_017534.6(*MYH2*):c.5673 + 1G > C	5′ ss mutation	Exon 39 skipping
Cardiomyopathy, dilated, 1A [26]	NM_170707.4(*LMNA*):c.356 + 1G > A	5′ ss mutation	Cryptic 5′ ss usage in exon 1, results in 32 bp deletion
Lynch syndrome 1 [27]	NM_000251.2(*MSH2*):c.1661 + 2 T > G	5′ ss mutation	Exon 10 skipping; Cryptic 5′ ss usage in exon 10, results in 82 bp deletion
Werner syndrome [28]	NM_000553.6(*WRN*):c.2732 + 5G > A	5′ ss mutation	Exon 22 skipping
Mineralocorticoid deficiency, isolated [29]	NM_018490.5(*LGR4*):c.618-1G > C	3′ ss mutation	Exon 6 skipping; Cryptic 3′ ss usage in exon 6, results in 24 bp deletion
Muscular dystrophy, limb-girdle, autosomal recessive type 26 [30]	NM_022361.5(*POPDC3*):c.486-1G > A	3′ ss mutation	Cryptic 3′ ss usage in exon 3, results in 52 bp deletion
Global developmental delay, progressive cerebellar atrophy [31]	NM_015047.3(*EMC1*):c.287-1G > A	3′ ss mutation	Intron 3 retention; Cryptic 3′ ss usage in intron 3, results in 91 bp insertion; Cryptic 3′ ss usage in exon 4, results in 24 bp deletion; Exon 4 skipping
Retinitis pigmentosa with myopathy [32]	NM_016026.4(*RDH11*):c.75-3C > A	3′ ss mutation	Exon 2 skipping
Retinitis pigmentosa, nonsyndromic, macular involvement [33]	NM_024649.4(*BBS1*):c.592-21A > T	BP mutation	Exon 8 skipping; Cryptic BP usage in exon 8, results in 30 bp deletion
Frontotemporal dementia, with parkinsonism linked to chromosome 17 [34]	NM_005910.5(*MAPT*):c.823-10G > T	PPT mutation	Increased exon 10 inclusion
Hypothyroidism, congenital [35]	NM_000453.3(*SLC5A5*):c.1326A > C, p.T442T	ESE disruption	Exon 11 and 12 skipping
Ciliary dyskinesia, primary [36]	NM_001195831.2(*CFAP57*):c.1762C > T, p.R588X	ESE disruption	Exon 11 skipping
Deafness, nonsyndromic 110 [37]	NM_004086.3(*COCH*):c.1093_1101del, p.S365_N367del	ESE disruption	Exon 11 skipping
Erythropoietic protoporphyria [38]	NM_000140.3(*FECH*):c.464-1169A > C	ESS disruption	156 bp pseudoexon inclusion between exons 4 and 5
Dysferlinopathy [39]	NM_003494.3(*DYSF*):c.1397 + 649C > T	5′ ss creation	66 bp pseudoexon inclusion between exons 15 and 16
Nephronophthisis, with congenital hepatic fibrosis [40]	NM_153240.5(*NPHP3*):c.2805C > T, p.G935G	5′ ss creation	Cryptic 5′ ss usage in exon 20, results in 80 bp deletion
Nemaline myopathy 8 [41]	NM_152393.4(*KLHL40*):c.*152G > T	5′ ss creation	Splicing of a 78 bp cryptic intron within the 3′ UTR
Developmental and epileptic encephalopathy 31 [42]	NM_001288739.1(*DNM1*):c.1197-8G > A	3′ ss creation	Cryptic 3′ ss usage, extends exon 10a by 6 bp upstream
Hemophilia B [43]	NM_000133.3(*F9*):c.278-1786_278-1785insLINE	5′ ss creation	137 bp pseudoexon inclusion between exons 3 and 4

Abbreviations: ss, splice site; bp, base pairs; BP, branchpoint; PPT, polypyrimidine tract; ESE, exonic splicing enhancer; ESS, exonic splicing silencer; UTR, untranslated region; LINE, long interspersed nuclear element.

On the other hand, variants that disrupt splicing enhancer or silencer elements are much more challenging to identify based on primary sequence analysis, given that they may initially appear as missense, synonymous or deep intronic variants [44]. Splicing enhancer and silencer elements are typically short (between 6 and 8 bp), highly degenerate and sometimes partially overlap [45]. To further complicate this problem, the activities of these regulatory elements may differ depending on where they are positioned along the pre-mRNA as well as the expression levels of their partner RBPs in a given cellular context [19, 46]. In light of these challenges, mutations that perturb splicing enhancer or silencer elements are often overlooked by clinical diagnostic workflows, yet they are still known to underlie many rare diseases [12, 35–38]. In a recently described case of congenital hypothyroidism, a patient was found to carry a homozygous synonymous variant in *SLC5A5* exon 11 [35]. This variant was predicted to reduce the binding affinity of splicing factor SRSF5 to a putative exonic splicing enhancer element in exon 11, and a subsequent minigene splicing reporter assay confirmed that this variant induced skipping of both exons 11 and 12 (Figure 2B).

Just as mutations can disrupt existing *cis* acting sequence elements involved in splicing regulation, mutations can also create new *cis* regulatory elements that can impact normal splicing patterns. In particular, mutations that activate cryptic splice sites have been previously reported in several cases of rare diseases [39–42]. For example, a recurrent *de novo* intronic variant upstream of *DNM1* exon 10a was recently reported in eight individuals diagnosed with developmental and epileptic encephalopathy, which is characterized by delayed development and epilepsy refractory to medical treatment [42]. The intronic variant was found to create a cryptic 3′ splice site that extends the beginning of exon 10a by 6 bp, leading to the insertion of 2 amino acids into the oligomerization domain of dynamin 1 (Figure 2C). Of note, the impact of pathogenic splice site-creating mutations is not limited to changes in the coding regions of transcripts, but extends to changes in the untranslated regions (UTRs) as well. Recently, a point mutation deep within the 3′ UTR of *KLHL40* was found to create a cryptic 5′ splice site in a patient diagnosed with nemaline myopathy [41]. This point mutation results in the removal of an aberrant 78 bp intron from the 3′ UTR and provokes nonsense mediated mRNA decay (NMD), resulting in lower levels of *KLHL40* expression (Figure 2D). Despite their known involvement in disease etiology, mutations that activate cryptic splice sites frequently elude detection in clinical practice as they are also difficult to identify from primary sequence alone. This challenge is further exacerbated by the possibility that such cryptic splice sites are only active under certain cellular contexts, such as tissue type or developmental stage [47].

Finally, a less appreciated yet significant contributor to *cis* acting splicing defects in rare diseases is structural variation. In particular, the insertion of transposable elements, such as Alu and LINE-1, has become increasingly known to cause various genetic disorders by interrupting normal splicing patterns [43, 48]. A recent example of this was reported in a previously undiagnosed patient with hemophilia B who carried a ~6 kb LINE-1 insertion in intron 3 of *F9* [43]. According to the results of a minigene splicing reporter assay, this LINE-1 insertion leads to the exonization of a 137 bp chimeric pseudoexon that uses a 5′ splice site within the inserted sequence and a pre-existing 3′ splice site located upstream of the insertion site (Figure 2E). It is likely that structural variants impacting pre-mRNA splicing remain underreported in rare disease cases, as current methods for clinical DNA sequencing have limited capacity to profile structural variation [49]. We anticipate that as new DNA sequencing technologies become adopted by clinical diagnostic workflows, we will gain a better understanding of the extent to which structural variation contributes to splicing dysregulation in disease.

### Transcriptomics as a tool for investigating splicing defects in rare diseases

In recent years, a number of studies have used RNA-seq as a complementary assay for rare disease investigation in cases where prior clinical genetic testing had been unrevealing [50–55]. Across these studies, RNA-seq has not only helped with ascertaining the regulatory consequences of candidate variants but has also revealed new candidate genes and variants that were missed by previous genetic analyses. In particular, RNA-seq has enabled high-throughput discovery of aberrant splicing events and their underlying genomic variants across patient transcriptomes and offers many benefits over RT-PCR and minigene methods, which only examine a single gene or variant. Despite these advantages, however, most clinical laboratories do not offer RNA-seq as an additional assay for molecular diagnosis of rare diseases [56].

**Figure 3:**
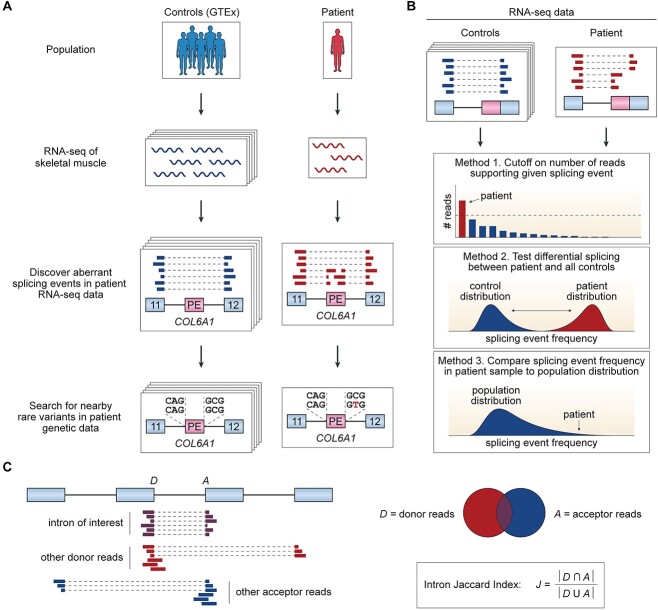
Detection of aberrant splicing events using RNA-seq data. (**A**) Cummings *et al*. analyzed RNA-seq data generated on muscle samples from 63 patients with rare muscle disorders as well as 184 GTEx controls. From their analysis, the authors identified a pseudoexon inclusion event in *COL6A1* that was present in four patients with collagen VI-related dystrophy and absent in GTEx controls and all other patients. Subsequent examination of patient genetic data revealed that this aberrant splicing event is caused by a nearby rare C > T splice site-creating mutation. (**B**) General categories of methods developed for calling aberrant splicing events from RNA-seq data. One method (Method 1) involves applying a combination of cutoffs to absolute and relative counts of RNA-seq reads supporting a given splicing event. Another method (Method 2) involves testing differential splicing between a single patient sample and all control samples. The third and more commonly used method (Method 3) requires fitting a distribution on the frequency of a given splicing event using all available samples (i.e. patient and control samples). This fitted distribution is then used to assess whether the splicing event frequency observed in a patient sample deviates significantly from expectation. (**C**) Schematic definition of the Intron Jaccard Index used in FRASER2. Abbreviations: RNA-seq, RNA sequencing; GTEx, Genotype-Tissue Expression; PE, pseudoexon.

One of the earliest applications of RNA-seq for rare disease diagnosis came from a pioneering study in 2017 from Cummings *et al*. [50], in which the authors profiled the muscle transcriptomes of 63 patients with rare muscle disorders and compared their RNA-seq data with 184 healthy control muscle samples from the Genotype-Tissue Expression (GTEx) project [57]. Among the 63 patients in this study, 13 had been previously diagnosed with variants expected to have transcriptional or post-transcriptional perturbations (e.g. altered gene expression or splicing patterns), whereas the remaining 50 undiagnosed cases either had a candidate splice-altering variant, a strong candidate gene, or no strong candidates based on prior genetic testing. The authors were able to achieve a diagnostic yield of 35% across previously undiagnosed individuals by discovering splicing aberrations in known disease genes that were unique to a small number of patients (i.e. outlier splicing events). For example, in four patients with collagen VI-related dystrophy, the authors found a splicing defect in *COL6A1* involving the inclusion of a 72 bp pseudoexon between exons 11 and 12 based on patient RNA-seq data (Figure 3A). Subsequent examination of patient genetic data revealed that this aberrant splicing event is induced by a deep intronic point mutation in *COL6A1* intron 11 that activates a cryptic 5′ splice site. *COL6A1* encodes a component of type VI collagen, and the observed mis-splicing event leads to the insertion of a 24 amino acid segment that disrupts Gly-X-Y repeat domains in the COL6A1 protein [47]. In the same year, a study from Kremer *et al*. also demonstrated the diagnostic potential of RNA-seq by analyzing fibroblast transcriptomes of 105 patients with suspected mitochondrial disease, among which 48 were undiagnosed based on prior exome sequencing analysis [51]. In their work, the authors reported a diagnostic yield of 10% across undiagnosed cases (5 out of 48), with three of the newly solved cases featuring splicing defects. Since these landmark publications in 2017, a rising number of other studies have also used RNA-seq to empower clinical variant interpretation and have reported diagnostic success rates ranging from 8 to 36% [52–55].

A major advance offered by more recent studies has been the development of robust statistical and computational methods for detecting outlier splicing events from patient RNA-seq data (Figure 3B). A commonly cited limitation of the method used by Cummings *et al*. for detecting splicing outliers is that it relied too heavily on *ad hoc* filters and thresholds [58, 59]. Specifically, Cummings *et al*. applied a combination of thresholds on absolute and relative counts of RNA-seq reads covering aberrant splice junctions [50], yet these thresholds were not data driven and statistical significance was not assessed. On the other hand, the study from Kremer *et al*. called splicing outliers in patient RNA-seq data derived from a cohort of individuals suspected of having mitochondrial disease by testing differential splicing between a single patient sample and all other samples via LeafCutter [51, 60]. Although the authors were able to control the false discovery rate of their findings, their approach for detecting outlier splicing events has limitations. Of note, LeafCutter uses a Dirichlet-Multinomial generalized linear model to compare the distributions of RNA-seq read counts across alternatively spliced exon–exon junctions between groups of samples. However, such a modeling framework is not well-suited for study designs involving one-versus-many comparisons (e.g. a single patient sample versus a group of healthy control samples), given that model parameter estimates for the group composed of a single sample would be statistically unreliable. Two relatively more recent approaches, LeafCutterMD and SPOT, address this issue by fitting a Dirichlet-Multinomial distribution directly to counts of RNA-seq reads spanning alternatively spliced exon–exon junctions using all samples, including patient and control samples [58, 61]. Subsequently, both methods identify individual samples whose RNA-seq read counts across the alternatively spliced exon–exon junctions deviate significantly from expectation based on the fitted distribution.

Although these two methods are certainly better suited for one-versus-many comparisons, they do not account for confounding variables, such as sequencing batch, that may influence quantifications of splicing [52, 62]. This limitation becomes especially critical in study designs where reference healthy controls are sequenced separately from patient samples. To address this issue, a 2019 study from Frésard *et al*. developed a method for calling aberrant splicing events that involves regressing out latent confounders from splicing quantifications via principal component analysis. Aberrant splicing events were subsequently called from corrected splicing metrics based on an absolute *z*-score cutoff [52]. More recently, Mertes *et al*. developed an algorithm called FRASER (Find RAre Splicing Events in RNA-seq) that provides a statistical test for identifying splicing outliers while controlling for latent confounders using a denoising autoencoder [59]. FRASER computes three different splicing metrics from input RNA-seq samples that correspond to different types of aberrant splicing patterns (i.e. 5′ splice site usage, 3′ splice site usage and intron retention) and uses a beta-binomial distribution to assess the statistical significance of candidate outlier events identified based on each of the three splicing metrics. Notably, a key advance offered by FRASER is the detection of outlier intron retention events from patient RNA-seq data, a feature that was overlooked by previous methods [50–52, 58, 61], which predominantly focused on exon skipping and alternative splice site usage. Applying FRASER to RNA-seq data generated on the patient cohort from Kremer *et al*., Mertes *et al*. were able to prioritize all three pathogenic splicing aberrations that were previously reported in the original study. More importantly, FRASER was also able to identify a new pathogenic splicing defect missed by Kremer *et al*. in an unsolved patient presenting with myopathic facies and arrhythmias. Specifically, Mertes *et al*. discovered a rare homozygous synonymous variant in *TAZ* exon 4, which created a cryptic 5′ splice site that removes 24 bp from *TAZ* transcripts and established the genetic diagnosis of Barth syndrome in this previously undiagnosed patient.

In a recent preprint, Mertes *et al*. describe FRASER2, an improved version of the original FRASER algorithm that substantially reduces the number of outlier splicing events reported in individual samples with minimal loss to sensitivity [63]. According to the authors, the three splicing metrics computed by FRASER are sensitive to sequencing depth and can lead to the discovery of outlier splicing events that may only have minor effects on the abundance of the canonical transcript isoform for the corresponding gene. FRASER2 overcomes this issue by introducing a new splicing metric termed the Intron Jaccard Index, which represents the proportion of reads spanning an intron of interest among all reads associated with either splice site of the intron (Figure 3C). The Intron Jaccard Index captures the former three metrics computed by FRASER and is more robust to variations in sequencing depth. Its use as the sole splicing metric in FRASER2 not only reduces runtime and computational resource requirements but also decreases the number of reported splicing outliers by nearly one order of magnitude compared with its predecessor.

Although it is clear that advances in statistical methods for detecting splicing outliers from patient RNA-seq data are paving the way for improved diagnostic yields in clinical settings, a considerable number of rare disease patients still remain undiagnosed after RNA-seq analysis [50–55]. To better maximize the utility of transcriptome sequencing for uncovering disease-causing splicing defects, we believe that several key considerations and challenges need to be addressed. First, it is important to acknowledge that the likelihood of discovering pathogenic splicing events from RNA-seq data of undiagnosed patients largely depends on how much is already known about potential causal genes underlying patient phenotypes. For example, for unsolved cases featuring autosomal recessive conditions in which one pathogenic allele has already been identified, there is a relatively high chance of finding a disease-relevant splicing defect affecting the second allele [50], particularly if there is a phenotypic match between the patient and the implicated disease. In contrast, for cases in which no candidate genes have been identified, or cases where patient phenotypes may be explained by a large number of potential candidate genes, more work is needed to distinguish clinically relevant splicing aberrations from secondary findings.

Related to this point, one area of clinical transcriptomics that demands more attention is the development of standard guidelines for filtering outlier splicing events revealed by RNA-seq analysis. The number of outlier events called in each patient RNA-seq sample can often be intractably large for manual review (i.e. on the order of thousands) [59, 63], and not all events are functionally deleterious or relevant to patient phenotypes. One possible strategy to reduce this search space is to filter for events found in genes with known or potential associations with patient phenotypes. To facilitate this approach, web tools and resources such as the Human Phenotype Ontology database [64] and AMELIE [65] can help identify and prioritize genes linked to a set of input phenotype terms. Subsequently, these genes can be used for filtering outlier splicing events called from patient RNA-seq data. Alternatively, investigators can also filter outlier splicing events based on their predicted effects on resulting protein products. Tools like ExonImpact [66] represent early efforts to prioritize alternative splicing events based on their predicted effects on protein secondary and tertiary structural features (e.g. whether an event removes a known post-translational modification site). Indeed, the effectiveness of such filtering strategies could vary based on what protein-level annotations are available for the corresponding genes. However, continued updates to protein annotation databases, such as UniProt [67] and Pfam [68], as well as protein-folding prediction tools, such as AlphaFold [69], are expected to improve predictions of how aberrant splicing events impact protein structure and function. In turn, these predictions can further help reduce the number of outlier splicing events needed for manual inspection and downstream functional characterization.

A more commonly used approach for filtering outlier splicing events involves applying different thresholds on the absolute change in splicing event frequency observed in patient RNA-seq data relative to frequencies observed in control RNA-seq samples. This filtering strategy typically rests on the assumption that splicing events involving large changes in frequency are more likely to have strong physiological impacts, whereas small changes tend to be enriched for statistical noise [50, 53, 59]. Although this approach is practical and easy to implement, it is not always clear as to what threshold is required to establish pathogenicity. Of note, aberrant splicing events with seemingly small changes in splicing event frequency could also have deleterious effects on the gene product and be disease-causing. For example, a 2019 study from Mohammadi *et al*. re-analyzed RNA-seq data generated on the patient cohort from Cummings *et al*., and the authors discovered a pathogenic splicing event in an unsolved patient with limb-girdle muscular dystrophy that was missed in the original study [70]. This splicing event involves the inclusion of a 116 bp pseudoexon between exons 7 and 8 of *DES*, induced by a deep intronic heterozygous point mutation in intron 7 that creates a cryptic 3′ splice site. Based on analysis of patient RNA-seq data, this pseudoexon appeared to be included in less than 1% of *DES* transcripts, and as a result, this event was filtered out from downstream analysis in Cummings *et al*. Yet Mohammadi *et al*. discovered that *DES* transcripts harboring this pseudoexon were efficiently degraded by NMD, resulting in reduced *DES* expression from the allele carrying the deep intronic point mutation. The second allele, which harbors a pathogenic missense variant and expresses normally spliced *DES* transcripts, was found to be expressed at much higher levels. Consequently, the presence of allelic imbalance resulted in a misleading appearance of low inclusion levels for the *DES* pseudoexon based on patient RNA-seq data.

One strategy to address this limitation is to analyze allele-specific splicing from RNA-seq data. Measurements of allele-specific splicing can directly capture *cis* acting genetic effects on splicing, including those induced by rare variants [71, 72]. In practice, analyzing allele-specific splicing uses RNA-seq reads that overlap a given splicing event as well as a heterozygous single nucleotide variant, which can be used to assign reads to individual alleles. However, most transcriptome profiling studies commonly rely on short RNA-seq reads, which are typically not long enough to phase individual splicing events. On the other hand, long-read RNA-seq platforms, such as Pacific Biosciences and Oxford Nanopore Technologies, can generate reads longer than 10 kb [73], thereby enabling linkage of individual splicing events to nearby or distant heterozygous sites. In a recent study, Glinos *et al*. generated long-read RNA-seq data on the Oxford Nanopore Technologies platform for 88 samples from GTEx tissues and cell lines, and they subsequently analyzed allele-specific transcript splicing patterns from their data [74]. In doing so, the authors were able to discover both common and rare genomic variants that impact splicing, including variants in genes with disease relevance such as *PPA2* and *NDUFS4*.

More broadly, we anticipate that long-read RNA-seq can bring many other benefits to the space of clinical transcriptomics. First, by revealing the full-length sequences of transcript molecules, long-read RNA-seq provides an opportunity to better assess the functional impact of aberrant splicing. This becomes especially important in contexts where a gene harboring a splicing defect is known to express multiple transcript isoforms [75]. Moreover, long-read RNA-seq can enhance transcriptome-wide detection of splicing events that have traditionally been difficult to characterize using short reads as a result of read length limitations or read mapping challenges [76–78]. For example, short-read RNA-seq is not well suited for detecting intron retention as individual short reads are not long enough to resolve entire introns, which are typically longer than 1 kb [17]. Supporting this point, several recent studies have demonstrated that retained introns discovered from long-read RNA-seq data are detected with low precision and recall using sample-matched short-read RNA-seq data [77, 79]. As variants causing intron retention are known to underlie rare diseases [31, 50, 51], improving the detection of intron retention events from RNA-seq data is critical for discovering pathogenic splice-altering variants.

Although long-read sequencing technologies hold great potential for revolutionizing transcriptomics, its adoption in clinical settings has largely been hampered by technological limitations, such as relatively low sequencing throughput, high error rates and increased data storage requirements [80]. Nonetheless, there have been rapid improvements made to the throughput and accuracy of long-read sequencing technologies [81], and we are now starting to witness the emergence of long-read RNA-seq in newer studies featuring unsolved cases of rare diseases, such as inherited cardiomyopathies [26, 82]. We can only anticipate that continued advancements made to long-read technologies will soon prompt the corresponding development of new computational methods for identifying and characterizing aberrant splicing events from long-read RNA-seq data.

Lastly, given the developmental and cell-type specific nature of both gene expression and splicing, selecting the disease-relevant tissue for RNA-seq is critical for detecting causal aberrant splicing events. In clinical settings, however, the primary disease-affected tissue may not be readily accessible for RNA-seq analysis, such as brain tissue from patients presenting with neurological disorders. Previous studies using transcriptome sequencing to facilitate rare disease diagnosis have traditionally relied on clinically accessible tissues and cell lines from patients for RNA-seq analysis, such as blood, fibroblasts, lymphoblastoid cell lines and muscle [50–55]. Nevertheless, as certain aberrant splicing events or the corresponding genes themselves may only be expressed in specific temporal or cellular contexts, it is not always clear as to which clinically accessible tissues, if any, are appropriate for RNA-seq. To address this issue, tools such as MAJIQ-CAT [83] and PAGE [53] can allow investigators to identify clinically accessible tissues whose total gene expression and splicing patterns resemble those observed in the primary disease-affected tissue based on public datasets, such as those from GTEx. Alternatively, deeper sequencing of clinically accessible tissues can provide a complementary means to enhance discovery of splicing defects in disease-relevant genes, even if those genes are poorly expressed in the sequenced tissues. Although this undoubtedly comes at the expense of higher sequencing costs, a recently developed web tool, MRSD, can help determine the minimum required sequencing depth needed to profile splicing patterns for a predefined set of genes in different clinically accessible tissues [84]. These approaches can guide selection of the optimal proxy tissue for RNA-seq that will maximize the likelihood of detecting aberrant splicing events in patient samples while considering sequencing costs. Furthermore, in cases where there does not exist a clinically accessible tissue that can serve as a suitable proxy, patient-derived induced pluripotent stem cells (iPSCs) can be differentiated into cell types of the disease-relevant tissue. Analyzing the transcriptomes of differentiated iPSCs can enable the detection of pathogenic splicing events that would otherwise be missed, as demonstrated by several previous studies [85, 86]. As protocols for differentiation become more efficient and accessible, we anticipate that the generation of previously inaccessible cell types for transcriptome profiling can facilitate the discovery of new pathogenic splicing aberrations in rare diseases.

### Computational prediction of genomic variants impacting pre-mRNA splicing

While analyzing patient RNA-seq data can directly reveal aberrant splicing events underlying disease phenotypes, identifying the genomic variants responsible for the observed splicing events may not always be entirely obvious. Most rare disease studies typically perform manual inspection of genomic regions surrounding outlier splicing events to propose candidate splice-altering variants. To aid this effort, new tools such as WATERSHED [61] and DROP [87] are allowing investigators to automatically prioritize nearby rare variants associated with splicing outliers, provided that genomes paired with transcriptomes are available from rare disease patients.

On the other hand, computational tools designed to predict splice-altering variants directly from genome sequence can also be used to reveal candidate variants underlying splicing outliers found in patient RNA-seq data. In fact, such prediction tools can still prove to be useful in settings where patient samples are not available for transcriptome profiling. Over the years, there has been a surge in bioinformatics tools designed for predicting variants that impact pre-mRNA splicing [88]. A core premise underlying the development of these tools is that there exists a ‘splicing code,’ or a set of features and associated rules that govern splicing outcomes of any pre-mRNA sequence in a given cellular context [89–93]. To this end, an increasing number of computational tools are using deep learning algorithms to identify *cis* level features from genome sequence that dictate splicing outcomes. Learned features of the ‘splicing code’ can then be leveraged to assess the impact of genomic variants on splicing outcomes.

**Figure 4:**
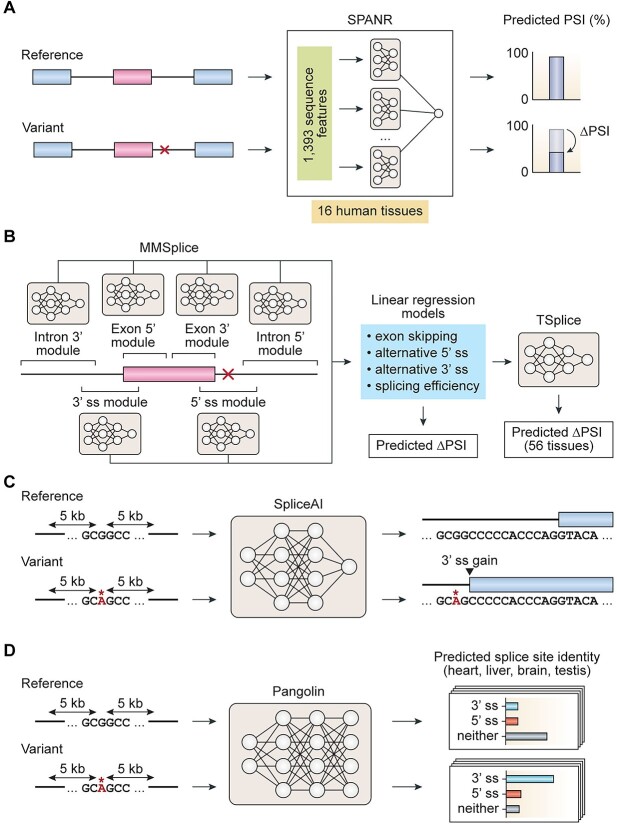
Deep learning-based tools for predicting splice-altering variants. (**A**) SPANR extracts 1393 sequence features surrounding a cassette exon of interest and uses an ensemble of Bayesian neural networks to model how these features cooperate or compete to influence cassette exon inclusion levels (measured in PSI) across 16 different human tissues. To quantify the impact of a single nucleotide variant on cassette exon inclusion, SPANR is applied to an exon sequence with and without the variant, and the largest absolute difference in predicted PSI values across all tissues is reported. (**B**) MMSplice consists of six neural networks, or modules, that score different sequences surrounding an exon of interest, including a 5′ splice site region (18 bp window from 5 bp upstream to 13 bp downstream of the 5′ splice site), a 3′ splice site region (53 bp window from 50 bp upstream to 3 bp downstream of the 3′ splice site), as well as 5′ and 3′ exonic and intronic sequences. Outputs from each module are combined with a linear regression model to score variant effects (in terms of ΔPSI) on exon skipping, alternative 5′ splice site usage, alternative 3′ splice site usage or splicing efficiency. To obtain tissue-specific predictions of ΔPSI, the MTSplice framework adjusts ΔPSI predictions made by MMSplice with TSplice, a separate neural network that predicts tissue-specific variations in splicing levels based on genome sequence. (**C** and **D**) Both SpliceAI and Pangolin consider a 10 kb window centered around a genomic position of interest and use deep neural networks to predict whether the position represents a 5′ splice site, 3′ splice site or neither. To assess whether a variant alters splicing, both tools are applied to a genomic sequence with and without the variant. The nucleotide position (within 50 bp of the queried variant) that is predicted to have the most substantial gain or loss of potential as a 5′ or 3′ splice site due to the variant is reported. Compared with SpliceAI, which makes tissue-agnostic predictions of splice-altering variants, Pangolin can predict splice-altering variants in four different tissues (heart, liver, brain, testis). Red crosses and asterisks represent mutations. Abbreviations: ss, splice site; PSI, percent spliced in; kb, kilobase.

One of the very first studies to showcase the power of machine learning for predicting splice-altering variants came from Xiong *et al*. in 2015 [91]. Building upon earlier computational models of alternative splicing regulation [89, 90], the authors developed a tool, SPANR, that can predict the impact of single nucleotide variants on cassette exon inclusion levels across multiple human tissues. To accomplish this, SPANR first extracts 1393 sequence features from a cassette exon of interest along with its flanking introns and exons (Figure 4A). SPANR subsequently uses an ensemble of Bayesian neural networks to model how individual features cooperate or compete to influence cassette exon inclusion levels across different human tissues. Specifically, exon inclusion levels are quantified as percent spliced in (PSI), a widely used metric representing the percentage of a gene’s transcripts that harbor a particular exon (or splice site) [94]. The authors trained SPANR using 10 689 cassette exons from NCBI RefSeq annotations [95], along with their corresponding PSI estimates from RNA-seq data of 16 human tissues [96]. To quantify the impact of a single nucleotide variant on cassette exon inclusion, SPANR is applied to an exon sequence with and without the variant, and the difference between predicted PSI values (ΔPSI) is reported for multiple different tissues. Finally, for each variant, SPANR reports the largest ΔPSI value across all tissues and considers any variant with a predicted absolute ΔPSI greater than 5% as splice-altering. In their paper, the authors demonstrated that SPANR can predict known splice-altering variants underlying different genetic disorders, such as spinal muscular atrophy and Lynch syndrome, and predicted ΔPSI values for these variants were found to agree well with measurements from RT-PCR. Although SPANR demonstrates a wide range of potential applications for rare disease investigation, it has several limitations that are worth noting. First, SPANR can only evaluate the splicing impact of single nucleotide variants within 300 bp of a cassette exon of interest. This means that indels and deep intronic mutations cannot be analyzed despite their known contribution to pathogenic splicing alterations (Table 1). Moreover, SPANR is limited by the scope of aberrant splicing outcomes that can be predicted from input variants, as it only assesses the impact of variants on cassette exon inclusion levels.

In 2019, Cheng *et al*. developed MMSplice (Modular Modeling of Splicing), a computational framework that is capable of predicting the effects of single nucleotide variants and indels on exon skipping as well as alternative splice site choice [97]. MMSplice is composed of six neural networks, each trained to score different splicing-relevant sequence regions surrounding an exon of interest (Figure 4B). These regions include a 5′ splice site region (18 bp window from 5 bp upstream to 13 bp downstream of the 5′ splice site), a 3′ splice site region (53 bp window from 50 bp upstream to 3 bp downstream of the 3′ splice site), as well as 5′ and 3′ exonic and intronic sequences. Specifically, the 5′ and 3′ splice site region modules were designed to distinguish bona fide splice sites from random sequences, using all 5′ and 3′ splice sites annotated in GENCODE v24 annotations [98] as a positive set during training. On the other hand, the 5′ and 3′ exonic and intronic sequence modules were designed to predict the relative usage of potential alternative 5′ and 3′ splice sites outside of canonical 5′ and 3′ splice sites. These modules were trained using data generated from a massively parallel reporter assay investigating the effects of millions of random 25-mer sequences on alternative splice site usage in HEK293 cells [92].

The authors then developed a series of linear regression models that use the outputs of each module to predict the impact of a genomic variant on different types of splicing outcomes in terms of ΔPSI. For example, the authors developed a linear regression model that uses module outputs to directly predict changes in exon skipping induced by genomic variants. This linear regression model was fitted using data from two massively parallel reporter assays, both of which quantified the impact of known human genomic variants from the Exome Aggregation Consortium on exon skipping [99, 100]. Similarly, the authors also developed a separate linear regression model that uses module outputs to predict changes in alternative splice site usage induced by variants. Quantifications of alternative 5′ and 3′ splice site usage based on RNA-seq of 1057 brain samples and 211 skin samples from GTEx were used to fit this regression model, together with genomic variants called from whole-genome sequencing data of GTEx donors [57]. MMSplice was ranked by the Fifth Critical Assessment of Genome Interpretation competition as being the best-performing method for predicting the impact of genomic variants on exon skipping [101], and scores from different MMSplice regression models have now become integrated with CADD, a well-known ensemble predictor of variant pathogenicity that is frequently used in clinical variant interpretation workflows [102, 103]. Recently, Cheng *et al*. developed MTSplice, a model that builds on the MMSplice framework to predict the splicing impact of genomic variants in a tissue-specific manner for 56 tissues [104]. Specifically, MTSplice achieves this by adjusting predictions made by MMSplice with TSplice (Tissue-specific Splicing), a separate deep neural network that predicts tissue-specific variations in splicing levels based on genome sequence. Of note, TSplice is trained on data from ASCOT, which catalogs PSI values for over 60 000 cassette exons across 56 human tissues based on RNA-seq data [105]. Applying MTSplice to *de novo* mutations from autism disorder simplex families [106], the authors were able to prioritize variants with brain-specific effects on splicing that were missed by MMSplice.

It is important to recognize, however, that there are still several limitations underlying the frameworks used by MMSplice and MTSplice. First, MMSplice was predominantly trained and evaluated on data from minigene splicing reporter assays. Despite their convenience and prevalent usage in validating splice-disrupting variants, minigenes do not entirely capture bona fide splicing patterns in human transcriptomes. For example, minigenes do not harbor full-length sequences of flanking introns due to size limitations and also do not capture local chromatin states, both of which are known to modulate splicing outcomes [107, 108]. Moreover, as the training data for MMSplice do not cover variants located more than 100 bp into introns, the authors do not recommend using their tool to predict the splicing impact of deep intronic variants, despite their known contributions to pathogenic splicing defects (Table 1).

At around the same time when MMSplice was developed, a deep learning-based method called SpliceAI was developed by Jaganathan *et al*. to predict 5′ and 3′ splice sites from an input pre-mRNA transcript sequence [109]. One of the underlying motivations behind developing SpliceAI was to enable the precise identification of genomic variants that activate cryptic splice sites, which are well known to play roles in rare disease etiology (Table 1) but commonly elude detection in clinical practice and by previous computational methods. SpliceAI uses a 32-layer deep residual neural network that considers all nucleotide positions within 50 bp of a given variant and returns the nucleotide position predicted to have the most substantial gain or loss of potential as a 5′ or 3′ splice site due to the variant (Figure 4C). To train SpliceAI, the authors used over 20 000 canonical protein-coding transcript annotations from GENCODE v24 and labeled each base within each transcript as either a 5′ splice site, 3′ splice site or a non-splice site. In this process, their model considers a 10 kb window (by default) centered around a given position and infers sequence features that define the position’s status as a 5′ splice site, 3′ splice site or neither.

A primary advantage of SpliceAI’s deep learning framework is that it can autonomously identify sequence features associated with splice site definition, including potentially novel features that have not been previously defined in the field. Given the predictive power of SpliceAI in detecting cryptic splice site mutations, it is no surprise that the tool has gained significant popularity in the rare disease research community, with over 900 citations over the past four years. Like MMSplice, SpliceAI has become integrated as a part of CADD, reflecting its perceived clinical utility in assessing variant pathogenicity. A current limitation of SpliceAI, however, is that it is not designed to predict variants that activate tissue-specific cryptic splice sites, which have been previously reported to underlie several cases of rare diseases [47, 110]. To address this, the authors note that their modeling framework can be further extended to make predictions of variants activating tissue-specific cryptic splice sites by training on splice junction annotations derived from RNA-seq of different human tissues. In addition, although SpliceAI claims predictive power in identifying cryptic splice site variants within 50 bp of annotated exons, model performance drops by nearly 2-fold when predicting splice site-creating variants deep within introns.

More recently, a 2022 study from Zeng *et al*. reported a new computational tool, Pangolin, that uses dilated convolutional neural networks to predict changes in splice site usage induced by genomic variants across four tissues, which include the heart, liver, brain and testis [111] (Figure 4D). Similar to the approach used by SpliceAI, Pangolin also considers a 10 kb window centered around a given genomic position and learns sequence features that explain the position’s identity as a 5′ splice site, 3′ splice site or non-splice site. A key difference from SpliceAI, however, is that Pangolin includes an additional neural network layer designated to predict splice site usage changes for different tissues. As predictions of splice site usage in multiple tissues may require large training sets, the authors trained Pangolin on data from four different species, including human, rhesus macaque, rat and mouse. Specifically, splice site usage was detected and quantified based on RNA-seq data of heart, liver, brain and testis from multiple samples per species [112], and this information, together with species-specific genome sequences, was used during model training. Notably, one major advance offered by Pangolin is that it allows users to predict epistatic effects of genomic variants on RNA splicing. This feature can be useful as the splicing impact of rare pathogenic variants may be modified through interactions with neighboring common variants [113]. To evaluate the accuracy to which Pangolin can predict the splicing impact of multiple mutations, the authors used their tool to predict PSI values based on a minigene splicing reporter assay featuring *FAS* exon 6 with different single-nucleotide variants or with a combination of multiple variants [113, 114]. The authors found that PSI values predicted by Pangolin showed Spearman’s correlations of 0.79 to 0.80 with experimentally determined PSI values for both single variants as well as combinations of variants.

While the steady rise in the number of computational methods designed to predict splice-altering variants is certainly an exciting development, a handful of challenges remain to be addressed in both research and clinical settings. For example, one area that requires more work is predicting tissue-specific effects of genomic variants on splicing. As revealed in performance evaluations of MTSplice and Pangolin, *in silico* predictions of tissue-specific splicing changes are still not entirely well correlated with splicing changes measured from RNA-seq data of the corresponding tissue [104, 111]. Moreover, it is important to recognize that pathogenic splice-altering variants can lead to many different splicing consequences, such as exon skipping, activation of alternative splice sites or intron retention (Table 1). Knowing the precise nature of variant-associated mis-splicing is important when making functional assessments of candidate splice-altering variants, yet most splice prediction tools typically predict a single splicing outcome (e.g. change in exon inclusion levels). To this end, investigators should run multiple computational tools designed to predict different variant-induced splicing consequences when possible. Lastly, in the space of clinical diagnostics, guidelines from the American College of Medical Genetics permit the use of computational predictions to support whether a variant impacts splicing, yet there are no standardized guidelines on what tools should be used and how they should be deployed. In particular, it is unclear what prediction score thresholds should be applied for each tool to establish potential evidence of pathogenicity. In a recent paper from the ClinGen Sequence Variant Interpretation Splicing Subgroup, the authors proposed several criteria for evaluating the performance of splicing prediction tools and calibrating tool thresholds to define pathogenic splicing variants [115]. With the rapid development of new splicing prediction tools that promise greater predictive power, having such standardized guidelines in place will be necessary for improving clinical classifications of splice-altering variants.

## CONCLUSION

This review highlights several major advances and improvements made to transcriptome sequencing analysis and predictive modeling that have accelerated our capacity to discover pathogenic variants impacting splicing. In clinical applications, diagnostic yields across rare diseases are expected to improve as we become more adept at uncovering splicing defects and their underlying genomic variants. Importantly, personalized splice-correction therapies are actively being developed to target splicing defects revealed by RNA-seq and splicing prediction tools [47, 116]. One such example of a personalized therapy is milasen, a splice-modulating antisense oligonucleotide drug that was tailored to a patient with neuronal ceroid lipofuscinosis, a rare and fatal neurodegenerative disease [117]. The patient carried a SINE-VNTR-Alu insertion in *MFSD8* that induced aberrant splicing of intron 6 based on RNA-seq analysis, and milasen was subsequently designed to correct intron 6 splicing. Within the span of a year after first contact with the patient, milasen was administered to the patient, who showed reduced frequency of seizures within months after treatment. Although there is certainly more work needed to refine experimental and computational approaches for studying variants that impact splicing, we anticipate that continued progress in this field will not only improve diagnostic yields but also pave the way for new precision therapies to be developed.

**Key Points**

- Variants occurring outside of canonical splice sites can induce pathogenic splicing events, yet they continue to be overlooked by clinical workflows for variant interpretation and genetic diagnosis.
- Improved statistical methods to detect outlier splicing events from RNA-seq data are enabling the discovery of novel pathogenic splicing variants in unsolved cases of rare diseases.
- Long-read RNA-seq provides new opportunities for detecting and interpreting splice-altering variants, as it provides a complete picture of splicing in full-length transcripts as well as phasing information connecting mis-spliced transcripts to disease-associated alleles.
- New deep learning-based computational tools are enabling investigators to predict splicing effects of variants in individual tissues using genome sequence alone.

## Author Biographies

**Robert Wang** is a PhD student affiliated with the Graduate Group in Genomics and Computational Biology at the University of Pennsylvania. He is interested in using long-read transcriptome sequencing to characterize the functional consequences of common and rare variants in human genomes.

**Ingo Helbig** is an Assistant Professor of Neurology at the University of Pennsylvania and a pediatric neurologist in the Division of Neurology at the Children’s Hospital of Philadelphia. His laboratory uses computational methods to understand the role of clinical and genetic factors in childhood epilepsies in order to facilitate precision medicine approaches.

**Andrew C. Edmondson** is an Assistant Professor of Pediatrics at the University of Pennsylvania and Children’s Hospital of Philadelphia. He is board certified in Clinical Genetics and Genomics, Clinical Biochemical Genetics, and Pediatrics. He is also the founding Director of the Congenital Disorders of Glycosylation (CDG) clinic at the Children’s Hospital of Philadelphia and leads a research program focused on elucidating the critical role of glycosylation in human neurodevelopment, understanding mechanisms of CDG pathophysiology and developing novel CDG-centric therapeutics.

**Lan Lin** is an Assistant Professor of Pathology and Laboratory Medicine at the University of Pennsylvania and Children’s Hospital of Philadelphia. Her laboratory develops cutting-edge long-read and single-cell technologies for studying transcriptomic variation in mammalian cells and applies these technologies to RNA-targeted diagnostics and therapeutics.

**Yi Xing** is the Francis West Lewis Endowed Chair, Founding Director of the Center for Computational and Genomic Medicine, Executive Director of the Department of Biomedical and Health Informatics at the Children’s Hospital of Philadelphia, and Professor of Pathology and Laboratory Medicine at the University of Pennsylvania.

## References

[1] Nguengang Wakap S, Lambert DM, Olry A, et al. Estimating cumulative point prevalence of rare diseases: analysis of the Orphanet database. Eur J Hum Genet 2020;28:165–73. 31527858 10.1038/s41431-019-0508-0 PMC6974615

[2] Haendel M, Vasilevsky N, Unni D, et al. How many rare diseases are there? Nat Rev Drug Discov 2020;19:77–8. 32020066 10.1038/d41573-019-00180-y PMC7771654

[3] Boycott KM, Rath A, Chong JX, et al. International cooperation to enable the diagnosis of all rare genetic diseases. Am J Hum Genet 2017;100:695–705. 28475856 10.1016/j.ajhg.2017.04.003 PMC5420351

[4] 100,000 Genomes Project Pilot Investigators, Smedley D, Smith KR, et al. 100,000 genomes pilot on rare-disease diagnosis in health care—preliminary report. N Engl J Med 2021;385:1868–80. 34758253 10.1056/NEJMoa2035790 PMC7613219

[5] Splinter K, Adams DR, Bacino CA, et al. Effect of genetic diagnosis on patients with previously undiagnosed disease. N Engl J Med 2018;379:2131–9. 30304647 10.1056/NEJMoa1714458 PMC6481166

[6] Wise AL, Manolio TA, Mensah GA, et al. Genomic medicine for undiagnosed diseases. Lancet 2019;394:533–40. 31395441 10.1016/S0140-6736(19)31274-7 PMC6709871

[7] Sheth N, Roca X, Hastings ML, et al. Comprehensive splice-site analysis using comparative genomics. Nucleic Acids Res 2006;34:3955–67. 16914448 10.1093/nar/gkl556 PMC1557818

[8] Ellingford JM, Ahn JW, Bagnall RD, et al. Recommendations for clinical interpretation of variants found in non-coding regions of the genome. Genome Med 2022;14:73. 35850704 10.1186/s13073-022-01073-3 PMC9295495

[9] Wang GS, Cooper TA. Splicing in disease: disruption of the splicing code and the decoding machinery. Nat Rev Genet 2007;8:749–61. 17726481 10.1038/nrg2164

[10] Pagani F, Baralle FE. Genomic variants in exons and introns: identifying the splicing spoilers. Nat Rev Genet 2004;5:389–96. 15168696 10.1038/nrg1327

[11] Park E, Pan Z, Zhang Z, et al. The expanding landscape of alternative splicing variation in human populations. Am J Hum Genet 2018;102:11–26. 29304370 10.1016/j.ajhg.2017.11.002 PMC5777382

[12] Richards S, Aziz N, Bale S, et al. Standards and guidelines for the interpretation of sequence variants: a joint consensus recommendation of the American College of Medical Genetics and Genomics and the Association for Molecular Pathology. Genet Med 2015;17:405–24. 25741868 10.1038/gim.2015.30 PMC4544753

[13] Rogalska ME, Vivori C, Valcárcel J. Regulation of pre-mRNA splicing: roles in physiology and disease, and therapeutic prospects. Nat Rev Genet 2023;24:251–69. 36526860 10.1038/s41576-022-00556-8

[14] Sharp PA . Split genes and RNA splicing. Cell 1994;77:805–15. 7516265 10.1016/0092-8674(94)90130-9

[15] Workman CT, Yin Y, Corcoran DL, et al. enoLOGOS: a versatile web tool for energy normalized sequence logos. Nucleic Acids Res 2005;33:W389–92. 15980495 10.1093/nar/gki439 PMC1160200

[16] Zhang P, Philippot Q, Ren W, et al. Genome-wide detection of human variants that disrupt intronic branchpoints. Proc Natl Acad Sci U S A 2022;119:e2211194119. 36306325 10.1073/pnas.2211194119 PMC9636908

[17] Piovesan A, Antonaros F, Vitale L, et al. Human protein-coding genes and gene feature statistics in 2019. BMC Res Notes 2019;12:315. 31164174 10.1186/s13104-019-4343-8 PMC6549324

[18] Wang Z, Burge CB. Splicing regulation: from a parts list of regulatory elements to an integrated splicing code. RNA 2008;14:802–13. 18369186 10.1261/rna.876308 PMC2327353

[19] Fu XD, Ares M, Jr. Context-dependent control of alternative splicing by RNA-binding proteins. Nat Rev Genet 2014;15:689–701. 25112293 10.1038/nrg3778 PMC4440546

[20] Pan Q, Shai O, Lee LJ, et al. Deep surveying of alternative splicing complexity in the human transcriptome by high-throughput sequencing. Nat Genet 2008;40:1413–5. 18978789 10.1038/ng.259

[21] Nilsen TW, Graveley BR. Expansion of the eukaryotic proteome by alternative splicing. Nature 2010;463:457–63. 20110989 10.1038/nature08909 PMC3443858

[22] Kelemen O, Convertini P, Zhang Z, et al. Function of alternative splicing. Gene 2013;514:1–30. 22909801 10.1016/j.gene.2012.07.083 PMC5632952

[23] Scotti MM, Swanson MS. RNA mis-splicing in disease. Nat Rev Genet 2016;17:19–32. 26593421 10.1038/nrg.2015.3 PMC5993438

[24] Stenson PD, Mort M, Ball EV, et al. The Human Gene Mutation Database: towards a comprehensive repository of inherited mutation data for medical research, genetic diagnosis and next-generation sequencing studies. Hum Genet 2017;136:665–77. 28349240 10.1007/s00439-017-1779-6 PMC5429360

[25] Cassini TA, Malicdan MCV, Macnamara EF, et al. MYH2-associated myopathy caused by a novel splice-site variant. Neuromuscul Disord 2023;33:257–62. 36774715 10.1016/j.nmd.2022.12.014 PMC10023425

[26] Sedaghat-Hamedani F, Rebs S, Kayvanpour E, et al. Genotype complements the phenotype: identification of the pathogenicity of an LMNA splice variant by nanopore long-read sequencing in a large DCM family. Int J Mol Sci 2022;23:12230. 10.3390/ijms232012230 PMC9602549 36293084

[27] Li J, Li Y, Ni H, et al. A novel splice-site mutation in MSH2 is associated with the development of Lynch syndrome. Front Oncol 2020;10:983. 32637358 10.3389/fonc.2020.00983 PMC7318799

[28] Atallah I, McCormick D, Good JM, et al. Partial lipodystrophy, severe dyslipidaemia and insulin resistant diabetes as early signs of Werner syndrome. J Clin Lipidol 2022;16:583–90. 35780059 10.1016/j.jacl.2022.06.004

[29] Lucas C, Sauter KS, Steigert M, et al. Loss of LGR4/GPR48 causes severe neonatal salt wasting due to disrupted WNT signaling altering adrenal zonation. J Clin Invest 2023;133:e164915. 10.1172/JCI164915 PMC9927937 36538378

[30] Zhang L, Li W, Weng Y, et al. A novel splice site variant in the POPDC3 causes autosomal recessive limb-girdle muscular dystrophy type 26. Clin Genet 2022;102:345–9. 35842834 10.1111/cge.14192

[31] Bryen SJ, Zhang K, Dziaduch G, et al. Compound heterozygous splicing variants expand the genotypic spectrum of EMC1-related disorders. Clin Genet 2023;103:553–9. 36799557 10.1111/cge.14311 PMC10101692

[32] Liu YD, Huang SS, Li M, et al. A new phenotype of syndromic retinitis pigmentosa with myopathy is caused by mutations in retinol dehydrogenase 11. Clin Genet 2022;101:448–53. 34988992 10.1111/cge.14108

[33] Fadaie Z, Whelan L, Dockery A, et al. BBS1 branchpoint variant is associated with non-syndromic retinitis pigmentosa. J Med Genet 2022;59:438–44.3391093210.1136/jmedgenet-2020-107626

[34] Olszewska DA, Fearon C, McGuigan C, et al. A clinical, molecular genetics and pathological study of a FTDP-17 family with a heterozygous splicing variant c.823-10G>T at the intron 9/exon 10 of the MAPT gene. Neurobiol Aging 2021;106:343.e1–8. 10.1016/j.neurobiolaging.2021.05.010 34274155

[35] Geysels RC, Bernal Barquero CE, Martín M, et al. Silent but not harmless: a synonymous SLC5A5 gene variant leading to dyshormonogenic congenital hypothyroidism. Front Endocrinol (Lausanne) 2022;13:868891. 35600585 10.3389/fendo.2022.868891 PMC9114739

[36] Bustamante-Marin XM, Horani A, Stoyanova M, et al. Mutation of CFAP57, a protein required for the asymmetric targeting of a subset of inner dynein arms in Chlamydomonas, causes primary ciliary dyskinesia. PLoS Genet 2020;16:e1008691. 32764743 10.1371/journal.pgen.1008691 PMC7444499

[37] Booth KT, Ghaffar A, Rashid M, et al. Novel loss-of-function mutations in COCH cause autosomal recessive nonsyndromic hearing loss. Hum Genet 2020;139:1565–74. 32562050 10.1007/s00439-020-02197-5 PMC7572817

[38] Chiara M, Primon I, Tarantini L, et al. Targeted resequencing of FECH locus reveals that a novel deep intronic pathogenic variant and eQTLs may cause erythropoietic protoporphyria (EPP) through a methylation-dependent mechanism. Genet Med 2020;22:35–43. 31273344 10.1038/s41436-019-0584-0

[39] Sun C, Xie Z, Cong L, et al. An in-frame pseudoexon activation caused by a novel deep-intronic variant in the dysferlin gene. Ann Clin Transl Neurol 2023;10:292–6. 36542547 10.1002/acn3.51716 PMC9930419

[40] Olinger E, Alawi IA, Al Riyami MS, et al. A discarded synonymous variant in NPHP3 explains nephronophthisis and congenital hepatic fibrosis in several families. Hum Mutat 2021;42:1221–8. 34212438 10.1002/humu.24251 PMC8434971

[41] Dofash LNH, Monahan GV, Servián-Morilla E, et al. A KLHL40 3′ UTR splice-altering variant causes milder NEM8, an under-appreciated disease mechanism. Hum Mol Genet 2023;32:1127–36. 10.1093/hmg/ddac272 36322148

[42] Parthasarathy S, Ruggiero SM, Gelot A, et al. A recurrent de novo splice site variant involving DNM1 exon 10a causes developmental and epileptic encephalopathy through a dominant-negative mechanism. Am J Hum Genet 2022;109:2253–69. 36413998 10.1016/j.ajhg.2022.11.002 PMC9748255

[43] Dericquebourg A, Fretigny M, Chatron N, et al. Whole F9 gene sequencing identified deep intronic variations in genetically unresolved hemophilia B patients. J Thromb Haemost 2023;21:828–37. 36696202 10.1016/j.jtha.2022.12.005

[44] Cartegni L, Chew SL, Krainer AR. Listening to silence and understanding nonsense: exonic mutations that affect splicing. Nat Rev Genet 2002;3:285–98. 11967553 10.1038/nrg775

[45] Wang J, Smith PJ, Krainer AR, et al. Distribution of SR protein exonic splicing enhancer motifs in human protein-coding genes. Nucleic Acids Res 2005;33:5053–62. 16147989 10.1093/nar/gki810 PMC1201331

[46] Lim KH, Ferraris L, Filloux ME, et al. Using positional distribution to identify splicing elements and predict pre-mRNA processing defects in human genes. Proc Natl Acad Sci U S A 2011;108:11093–8. 21685335 10.1073/pnas.1101135108 PMC3131313

[47] Bolduc V, Foley AR, Solomon-Degefa H, et al. A recurrent COL6A1 pseudoexon insertion causes muscular dystrophy and is effectively targeted by splice-correction therapies, *JCI* Insight 2019;4:e124403. 10.1172/jci.insight.124403 PMC6483063 30895940

[48] Payer LM, Burns KH. Transposable elements in human genetic disease. Nat Rev Genet 2019;20:760–72. 31515540 10.1038/s41576-019-0165-8

[49] Miller DE, Sulovari A, Wang T, et al. Targeted long-read sequencing identifies missing disease-causing variation. Am J Hum Genet 2021;108:1436–49. 34216551 10.1016/j.ajhg.2021.06.006 PMC8387463

[50] Cummings BB, Marshall JL, Tukiainen T, et al. Improving genetic diagnosis in Mendelian disease with transcriptome sequencing. Sci Transl Med 2017;9:eaal5209. 10.1126/scitranslmed.aal5209 PMC5548421 28424332

[51] Kremer LS, Bader DM, Mertes C, et al. Genetic diagnosis of Mendelian disorders via RNA sequencing. Nat Commun 2017;8:15824. 28604674 10.1038/ncomms15824 PMC5499207

[52] Frésard L, Smail C, Ferraro NM, et al. Identification of rare-disease genes using blood transcriptome sequencing and large control cohorts. Nat Med 2019;25:911–9. 31160820 10.1038/s41591-019-0457-8 PMC6634302

[53] Gonorazky HD, Naumenko S, Ramani AK, et al. Expanding the boundaries of RNA sequencing as a diagnostic tool for rare Mendelian disease. Am J Hum Genet 2019;104:466–83. 30827497 10.1016/j.ajhg.2019.01.012 PMC6407525

[54] Lee H, Huang AY, Wang LK, et al. Diagnostic utility of transcriptome sequencing for rare Mendelian diseases. Genet Med 2020;22:490–9. 31607746 10.1038/s41436-019-0672-1 PMC7405636

[55] Murdock DR, Dai H, Burrage LC, et al. Transcriptome-directed analysis for Mendelian disease diagnosis overcomes limitations of conventional genomic testing. J Clin Invest 2021;131:e141500. 10.1172/JCI141500 PMC7773386 33001864

[56] Montgomery SB, Bernstein JA, Wheeler MT. Toward transcriptomics as a primary tool for rare disease investigation. Cold Spring Harb Mol Case Stud 2022;8:a006198. 10.1101/mcs.a006198 PMC8958920 35217565

[57] GTEx Consortium. Human genomics. The Genotype-Tissue Expression (GTEx) pilot analysis: multitissue gene regulation in humans. Science 2015;348:648–60. 25954001 10.1126/science.1262110 PMC4547484

[58] Jenkinson G, Li YI, Basu S, et al. LeafCutterMD: an algorithm for outlier splicing detection in rare diseases. Bioinformatics 2020;36:4609–15. 32315392 10.1093/bioinformatics/btaa259 PMC7750945

[59] Mertes C, Scheller IF, Yépez VA, et al. Detection of aberrant splicing events in RNA-seq data using FRASER. Nat Commun 2021;12:529. 33483494 10.1038/s41467-020-20573-7 PMC7822922

[60] Li YI, Knowles DA, Humphrey J, et al. Annotation-free quantification of RNA splicing using LeafCutter. Nat Genet 2018;50:151–8. 29229983 10.1038/s41588-017-0004-9 PMC5742080

[61] Ferraro NM, Strober BJ, Einson J, et al. Transcriptomic signatures across human tissues identify functional rare genetic variation. Science 2020;369:eaaz5900. 10.1126/science.aaz5900 PMC7646251 32913073

[62] Slaff B, Radens CM, Jewell P, et al. MOCCASIN: a method for correcting for known and unknown confounders in RNA splicing analysis. Nat Commun 2021;12:3353. 34099673 10.1038/s41467-021-23608-9 PMC8184769

[63] Scheller IF, Lutz K, Mertes C, et al. Improved detection of aberrant splicing using the intron Jaccard index. *medRxiv* 2023. 10.1016/j.ajhg.2023.10.014 PMC10716352 38006880

[64] Köhler S, Carmody L, Vasilevsky N, et al. Expansion of the Human Phenotype Ontology (HPO) knowledge base and resources. Nucleic Acids Res 2019;47:D1018–27. 30476213 10.1093/nar/gky1105 PMC6324074

[65] Birgmeier J, Haeussler M, Deisseroth CA, et al. AMELIE speeds Mendelian diagnosis by matching patient phenotype and genotype to primary literature. Sci Transl Med 2020;12:eaau9113. 10.1126/scitranslmed.aau9113 PMC9366928 32434849

[66] Li M, Feng W, Zhang X, et al. ExonImpact: prioritizing pathogenic alternative splicing events. Hum Mutat 2017;38:16–24. 27604408 10.1002/humu.23111 PMC5390777

[67] UniProt Consortium. UniProt: the universal protein knowledgebase in 2023. Nucleic Acids Res 2023;51:D523–31. 36408920 10.1093/nar/gkac1052 PMC9825514

[68] Mistry J, Chuguransky S, Williams L, et al. Pfam: the protein families database in 2021. Nucleic Acids Res 2021;49:D412–9. 33125078 10.1093/nar/gkaa913 PMC7779014

[69] Jumper J, Evans R, Pritzel A, et al. Highly accurate protein structure prediction with AlphaFold. Nature 2021;596:583–9. 34265844 10.1038/s41586-021-03819-2 PMC8371605

[70] Mohammadi P, Castel SE, Cummings BB, et al. Genetic regulatory variation in populations informs transcriptome analysis in rare disease. Science 2019;366:351–6. 31601707 10.1126/science.aay0256 PMC6814274

[71] Demirdjian L, Xu Y, Bahrami-Samani E, et al. Detecting allele-specific alternative splicing from population-scale RNA-seq data. Am J Hum Genet 2020;107:461–72. 32781045 10.1016/j.ajhg.2020.07.005 PMC7477012

[72] Amoah K, Hsiao YE, Bahn JH, et al. Allele-specific alternative splicing and its functional genetic variants in human tissues. Genome Res 2021;31:359–71. 33452016 10.1101/gr.265637.120 PMC7919445

[73] Amarasinghe SL, Su S, Dong X, et al. Opportunities and challenges in long-read sequencing data analysis. Genome Biol 2020;21:30. 32033565 10.1186/s13059-020-1935-5 PMC7006217

[74] Glinos DA, Garborcauskas G, Hoffman P, et al. Transcriptome variation in human tissues revealed by long-read sequencing. Nature 2022;608:353–9. 35922509 10.1038/s41586-022-05035-y PMC10337767

[75] Wright DJ, Hall NAL, Irish N, et al. Long read sequencing reveals novel isoforms and insights into splicing regulation during cell state changes. BMC Genomics 2022;23:42. 35012468 10.1186/s12864-021-08261-2 PMC8744310

[76] Broseus L, Ritchie W. Challenges in detecting and quantifying intron retention from next generation sequencing data. Comput Struct Biotechnol J 2020;18:501–8. 32206209 10.1016/j.csbj.2020.02.010 PMC7078297

[77] David JK, Maden SK, Wood MA, et al. Retained introns in long RNA-seq reads are not reliably detected in sample-matched short reads. Genome Biol 2022;23:240. 36369064 10.1186/s13059-022-02789-6 PMC9652823

[78] Lanciano S, Cristofari G. Measuring and interpreting transposable element expression. Nat Rev Genet 2020;21:721–36. 32576954 10.1038/s41576-020-0251-y

[79] Gao Y, Wang F, Wang R, et al. ESPRESSO: robust discovery and quantification of transcript isoforms from error-prone long-read RNA-seq data. Sci Adv 2023;9:eabq5072. 36662851 10.1126/sciadv.abq5072 PMC9858503

[80] Byrne A, Cole C, Volden R, et al. Realizing the potential of full-length transcriptome sequencing. Philos Trans R Soc Lond B Biol Sci 2019;374:20190097. 31587638 10.1098/rstb.2019.0097 PMC6792442

[81] Sereika M, Kirkegaard RH, Karst SM, et al. Oxford Nanopore R10.4 long-read sequencing enables the generation of near-finished bacterial genomes from pure cultures and metagenomes without short-read or reference polishing. Nat Methods 2022;19:823–6. 35789207 10.1038/s41592-022-01539-7 PMC9262707

[82] Dainis A, Tseng E, Clark TA, et al. Targeted long-read RNA sequencing demonstrates transcriptional diversity driven by splice-site variation in MYBPC3. Circ Genom Precis Med 2019;12:e002464. 31112421 10.1161/CIRCGEN.119.002464

[83] Aicher JK, Jewell P, Vaquero-Garcia J, et al. Mapping RNA splicing variations in clinically accessible and nonaccessible tissues to facilitate Mendelian disease diagnosis using RNA-seq. Genet Med 2020;22:1181–90. 32225167 10.1038/s41436-020-0780-y PMC7335339

[84] Rowlands CF, Taylor A, Rice G, et al. MRSD: a quantitative approach for assessing suitability of RNA-seq in the investigation of mis-splicing in Mendelian disease. Am J Hum Genet 2022;109:210–22. 35065709 10.1016/j.ajhg.2021.12.014 PMC8874219

[85] Bonder MJ, Smail C, Gloudemans MJ, et al. Identification of rare and common regulatory variants in pluripotent cells using population-scale transcriptomics. Nat Genet 2021;53:313–21. 33664507 10.1038/s41588-021-00800-7 PMC7944648

[86] Mullin NK, Voigt AP, Cooke JA, et al. Patient derived stem cells for discovery and validation of novel pathogenic variants in inherited retinal disease. Prog Retin Eye Res 2021;83:100918. 33130253 10.1016/j.preteyeres.2020.100918 PMC8559964

[87] Yépez VA, Gusic M, Kopajtich R, et al. Clinical implementation of RNA sequencing for Mendelian disease diagnostics. Genome Med 2022;14:38. 35379322 10.1186/s13073-022-01019-9 PMC8981716

[88] Rowlands CF, Baralle D, Ellingford JM. Machine learning approaches for the prioritization of genomic variants impacting pre-mRNA splicing. Cells 2019;8:1513. 10.3390/cells8121513 PMC6953098 31779139

[89] Barash Y, Calarco JA, Gao W, et al. Deciphering the splicing code. Nature 2010;465:53–9. 20445623 10.1038/nature09000

[90] Zhang C, Frias MA, Mele A, et al. Integrative modeling defines the Nova splicing-regulatory network and its combinatorial controls. Science 2010;329:439–43. 20558669 10.1126/science.1191150 PMC3412410

[91] Xiong HY, Alipanahi B, Lee LJ, et al. RNA splicing. The human splicing code reveals new insights into the genetic determinants of disease. Science 2015;347:1254806. 25525159 10.1126/science.1254806 PMC4362528

[92] Rosenberg AB, Patwardhan RP, Shendure J, et al. Learning the sequence determinants of alternative splicing from millions of random sequences. Cell 2015;163:698–711. 26496609 10.1016/j.cell.2015.09.054

[93] Zhang Z, Pan Z, Ying Y, et al. Deep-learning augmented RNA-seq analysis of transcript splicing. Nat Methods 2019;16:307–10. 30923373 10.1038/s41592-019-0351-9 PMC7605494

[94] Katz Y, Wang ET, Airoldi EM, et al. Analysis and design of RNA sequencing experiments for identifying isoform regulation. Nat Methods 2010;7:1009–15. 21057496 10.1038/nmeth.1528 PMC3037023

[95] Pruitt KD, Tatusova T, Maglott DR. NCBI reference sequences (RefSeq): a curated non-redundant sequence database of genomes, transcripts and proteins. Nucleic Acids Res 2007;35:D61–5. 17130148 10.1093/nar/gkl842 PMC1716718

[96] Barbosa-Morais NL, Irimia M, Pan Q, et al. The evolutionary landscape of alternative splicing in vertebrate species. Science 2012;338:1587–93. 23258890 10.1126/science.1230612

[97] Cheng J, Nguyen TYD, Cygan KJ, et al. MMSplice: modular modeling improves the predictions of genetic variant effects on splicing. Genome Biol 2019;20:48. 30823901 10.1186/s13059-019-1653-z PMC6396468

[98] Harrow J, Frankish A, Gonzalez JM, et al. GENCODE: the reference human genome annotation for the ENCODE Project. Genome Res 2012;22:1760–74. 22955987 10.1101/gr.135350.111 PMC3431492

[99] Adamson SI, Zhan L, Graveley BR. Vex-seq: high-throughput identification of the impact of genetic variation on pre-mRNA splicing efficiency. Genome Biol 2018;19:71. 29859120 10.1186/s13059-018-1437-x PMC5984807

[100] Cheung R, Insigne KD, Yao D, et al. A multiplexed assay for exon recognition reveals that an unappreciated fraction of rare genetic variants cause large-effect splicing disruptions. Mol Cell 2019;73:183–194.e8. 30503770 10.1016/j.molcel.2018.10.037 PMC6599603

[101] Mount SM, Avsec Z, Carmel L, et al. Assessing predictions of the impact of variants on splicing in CAGI5. Hum Mutat 2019;40:1215–24. 31301154 10.1002/humu.23869 PMC6744318

[102] Rentzsch P, Witten D, Cooper GM, et al. CADD: predicting the deleteriousness of variants throughout the human genome. Nucleic Acids Res 2019;47:D886–94. 30371827 10.1093/nar/gky1016 PMC6323892

[103] Rentzsch P, Schubach M, Shendure J, et al. CADD-splice-improving genome-wide variant effect prediction using deep learning-derived splice scores. Genome Med 2021;13:31. 33618777 10.1186/s13073-021-00835-9 PMC7901104

[104] Cheng J, Çelik MH, Kundaje A, et al. MTSplice predicts effects of genetic variants on tissue-specific splicing. Genome Biol 2021;22:94. 33789710 10.1186/s13059-021-02273-7 PMC8011109

[105] Ling JP, Wilks C, Charles R, et al. ASCOT identifies key regulators of neuronal subtype-specific splicing. Nat Commun 2020;11:137. 31919425 10.1038/s41467-019-14020-5 PMC6952364

[106] Zhou J, Park CY, Theesfeld CL, et al. Whole-genome deep-learning analysis identifies contribution of noncoding mutations to autism risk. Nat Genet 2019;51:973–80. 31133750 10.1038/s41588-019-0420-0 PMC6758908

[107] Fox-Walsh KL, Dou Y, Lam BJ, et al. The architecture of pre-mRNAs affects mechanisms of splice-site pairing. Proc Natl Acad Sci U S A 2005;102:16176–81. 16260721 10.1073/pnas.0508489102 PMC1283478

[108] Kolasinska-Zwierz P, Down T, Latorre I, et al. Differential chromatin marking of introns and expressed exons by H3K36me3. Nat Genet 2009;41:376–81. 19182803 10.1038/ng.322 PMC2648722

[109] Jaganathan K, Kyriazopoulou Panagiotopoulou S, McRae JF, et al. Predicting splicing from primary sequence with deep learning. Cell 2019;176:535–548.e24. 30661751 10.1016/j.cell.2018.12.015

[110] Albert S, Garanto A, Sangermano R, et al. Identification and rescue of splice defects caused by two neighboring deep-intronic ABCA4 mutations underlying Stargardt disease. Am J Hum Genet 2018;102:517–27. 29526278 10.1016/j.ajhg.2018.02.008 PMC5985352

[111] Zeng T, Li YI. Predicting RNA splicing from DNA sequence using Pangolin. Genome Biol 2022;23:103. 35449021 10.1186/s13059-022-02664-4 PMC9022248

[112] Cardoso-Moreira M, Halbert J, Valloton D, et al. Gene expression across mammalian organ development. Nature 2019;571:505–9. 31243369 10.1038/s41586-019-1338-5 PMC6658352

[113] Baeza-Centurion P, Miñana B, Schmiedel JM, et al. Combinatorial genetics reveals a scaling law for the effects of mutations on splicing. Cell 2019;176:549–563.e23. 30661752 10.1016/j.cell.2018.12.010

[114] Julien P, Miñana B, Baeza-Centurion P, et al. The complete local genotype-phenotype landscape for the alternative splicing of a human exon. Nat Commun 2016;7:11558. 27161764 10.1038/ncomms11558 PMC4866304

[115] Walker LC, de la Hoya M, Wiggins GA, et al. Application of the ACMG/AMP framework to capture evidence relevant to predicted and observed impact on splicing: recommendations from the Clingen SVI Splicing Subgroup. *medRxiv* 2023. 10.1016/j.ajhg.2023.06.002 PMC10357475 37352859

[116] Kumar R, Corbett MA, Smith NJC, et al. Oligonucleotide correction of an intronic TIMMDC1 variant in cells of patients with severe neurodegenerative disorder. NPJ Genom Med 2022;7:9. 35091571 10.1038/s41525-021-00277-7 PMC8799713

[117] Kim J, Hu C, Moufawad El Achkar C, et al. Patient-customized oligonucleotide therapy for a rare genetic disease. N Engl J Med 2019;381:1644–52. 31597037 10.1056/NEJMoa1813279 PMC6961983

